# Supplementary material for: Fusaric acid mediates the assembly of disease-suppressive rhizosphere microbiota via induced shifts in plant root exudates
Source: Nat Commun. 2024 Jun 15;15:5125. doi: 10.1038/s41467-024-49218-9 (PMC11180119; doi:10.1038/s41467-024-49218-9)
Supplement: Supplementary file 1 — Supplementary Information [file 41467_2024_49218_MOESM1_ESM.pdf]

Supplementary Information for

Fusaric acid mediates the assembly of disease-suppressive rhizosphere microbiota via induced shifts in plant root exudates

Supplementary Figures

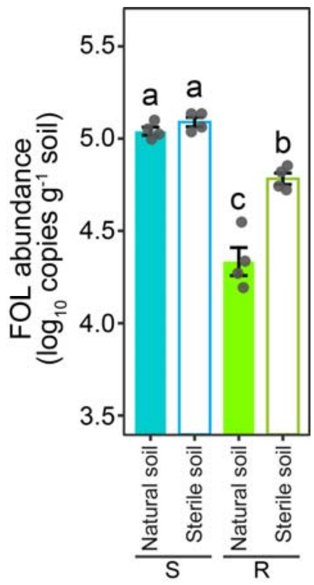

Supplementary Fig. 1 FOL abundance in the rhizosphere of tomato plants grown in natural soil or sterile soil. S, susceptible cultivar D72; R, resistant cultivar Z19; FOL, *F. oxysporum* f. sp. *lycopersici*. Data are shown as mean ± SEM (*n*=4). Different letters represent significant differences between treatments (Tukey's HSD test; $P < 0.05$).

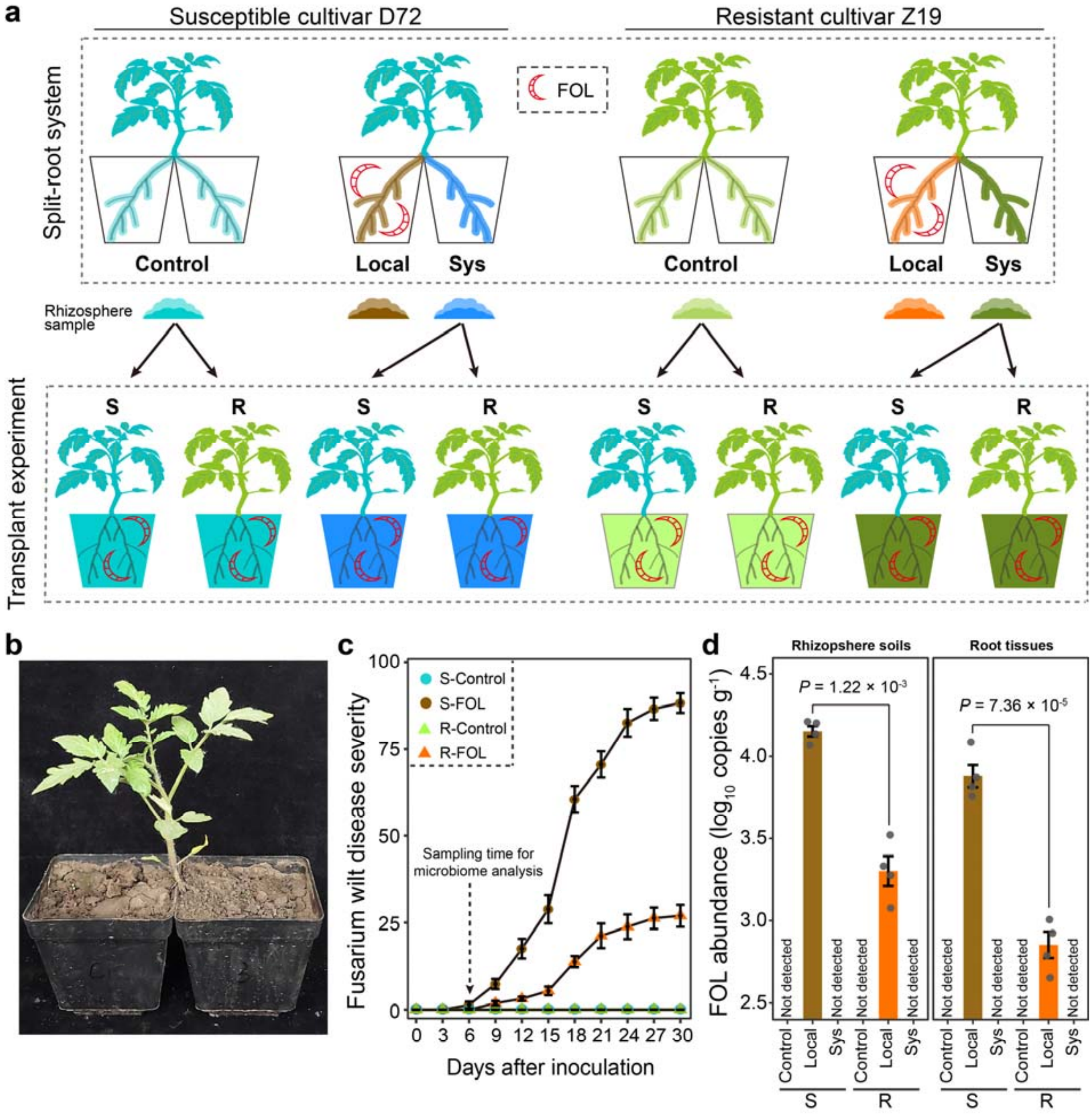

Supplementary Fig. 2 Experimental set-up of the split-root system and the rhizosphere transplant experiment, Fusarium wilt disease progression and FOL abundance in the split-root system. a Experiment set-up of the split-root system and rhizosphere transplant experiment. In the split-root system, tomato plants were transplanted into two pots, with part of the root system grown in each pot. One part of root system was inoculated with FOL (local), and the other part was left non-inoculated (systemic). For the control treatment, both sides of the root system were untreated. For the rhizosphere transplant experiment, tomato plants were grown in sterile soils mixed with rhizosphere samples from the split-root experiment. S, susceptible cultivar D72; R, resistant cultivar Z19. **b** The photo shows a tomato seedling grown in the split-root system. **c** Fusarium wilt disease progression (day 0 to 30 after inoculation) across treatments. **d** Abundance of FOL in the tomato rhizosphere and in root tissues. FOL, *F. oxysporum* f. sp. *lycopersici*; Sys, systemic. For **c** and **d**, data are shown as mean ± SEM (n=4). P values were determined through two-sided Welch's t-tests.

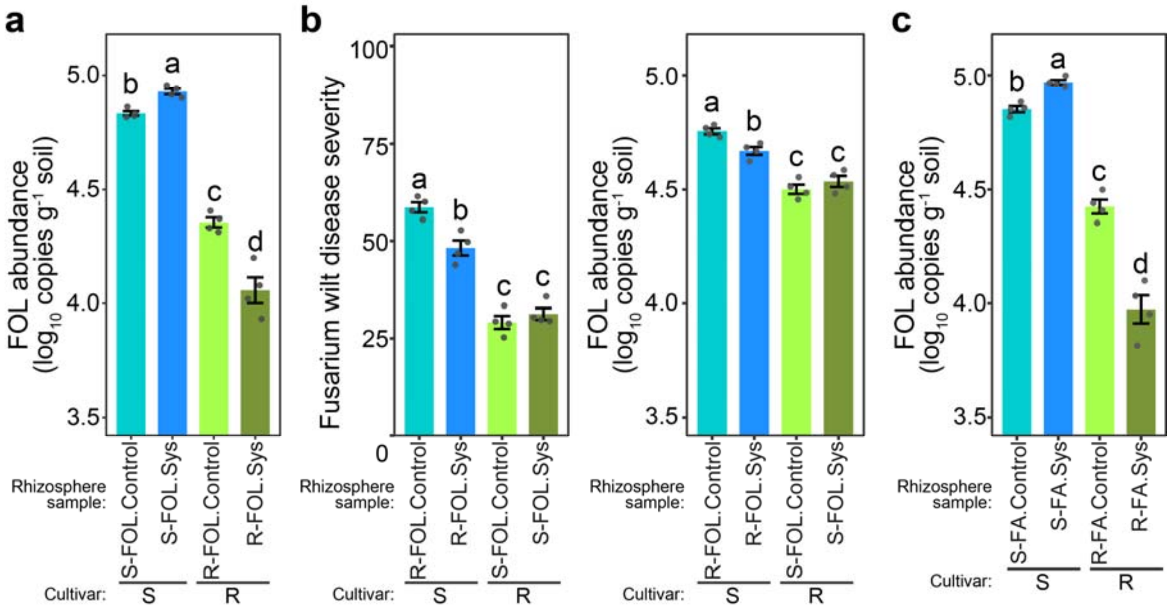

Supplementary Fig. 3 Rhizosphere FOL abundance in tomato plants in the rhizosphere transplant experiment. a FOL abundance in the rhizosphere of tomato plants in the transplant experiment testing the effects of FOL on rhizosphere suppressiveness. S, susceptible cultivar D72; R, resistant cultivar Z19. **b** Fusarium wilt disease severity and rhizosphere FOL abundance in the rhizosphere transplant experiment testing the effect(s) of FOL on the tomato rhizosphere disease suppressiveness. Susceptible and resistant cultivar were cross-transplanted with the rhizosphere sample from the split-root system. **c** FOL abundance in the rhizosphere of tomato plants in the transplant experiment testing the effects of FA amendment on rhizosphere suppressiveness. FOL, *F. oxysporum* f. sp. *lycopersici*; FA, fusaric acid; Sys, systemic. Data are shown as mean ± SEM (n=4). Different letters represent significant differences between treatments (Tukey's HSD test; $P < 0.05$).

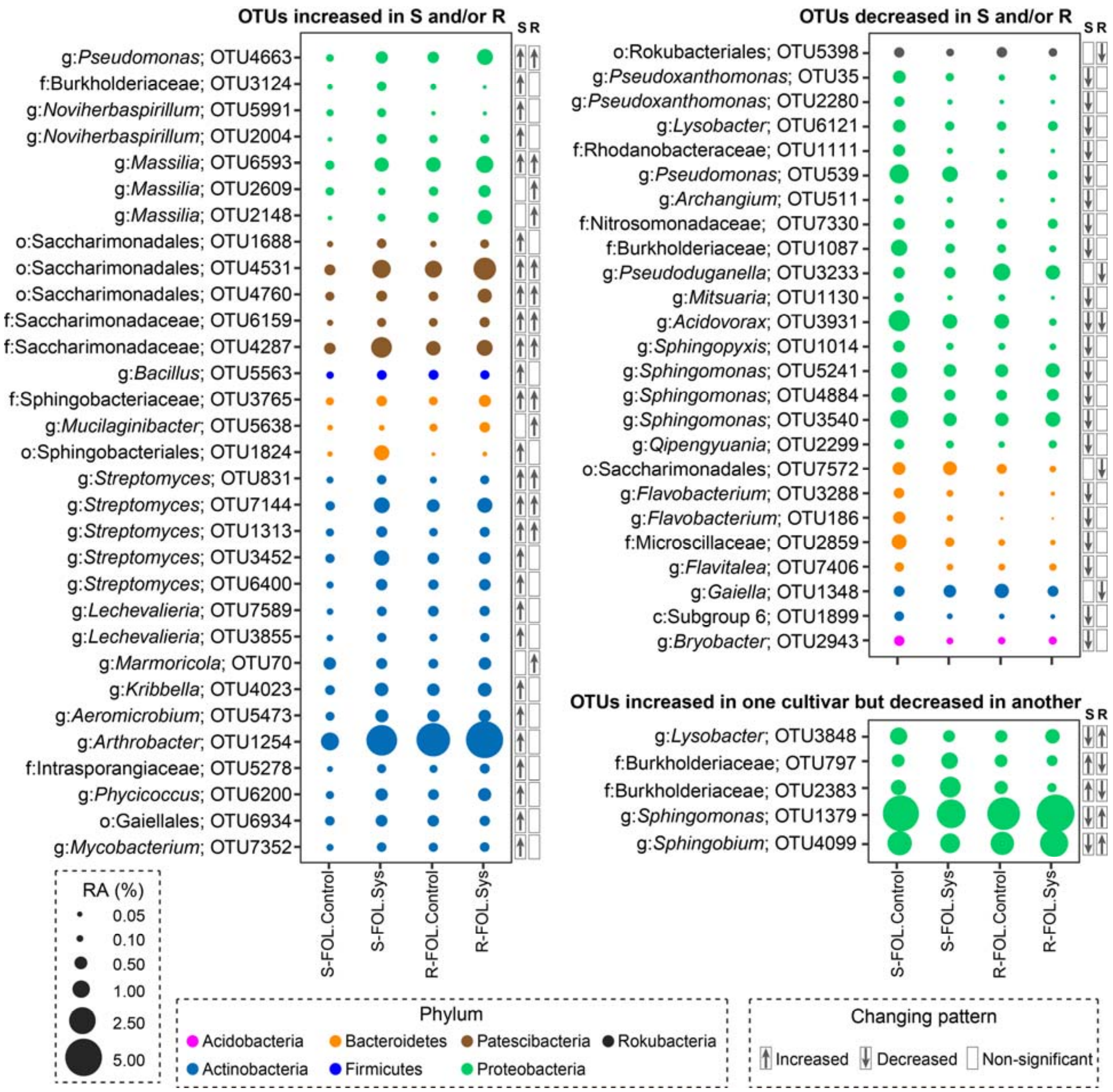

Supplementary Fig. 4 Relative abundance (RA) of bacterial OTUs that were systemically altered by FOL. The nearest taxonomy assignment for each OTU is shown on the left side (c, class; f, family; o, order; g, genus). The size of the dot corresponds to the RA of each OTU and the color depicts the phylum. The arrows on the right side of the panels indicated the changing pattern of each OTU. For each tomato cultivar, OTUs altered by FOL were identified using the Wald significance test in the DESeq2 package (two-sided Wald test, Benjamini-Hochberg adjusted $P < 0.01$). In the split-root system, one part of the root system was inoculated with FOL (local), and the other part was untreated (systemic). For the control treatment, both sides of the root system were untreated. S, susceptible cultivar D72; R, resistant cultivar Z19; Sys, systemic; FOL, *F. oxysporum* f. sp. *lycopersici*.

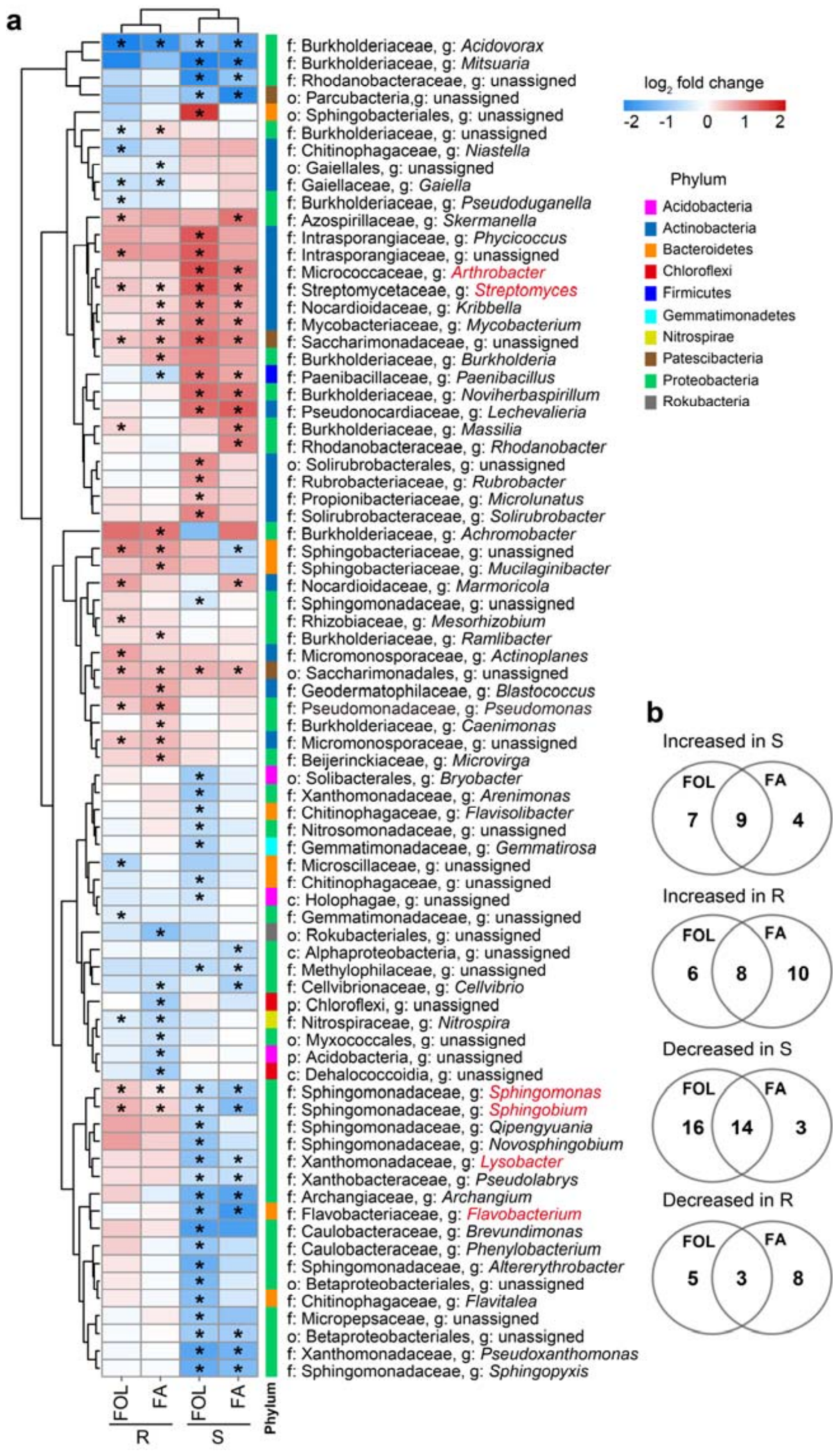

Supplementary Fig. 5 Comparison of bacterial genera altered by FOL and FA. a Heatmap depicting changes in bacterial genera altered by FOL and FA. Taxonomic affiliation of the genus: c, class; f, family; o, order; g, genus. Data (log_2 fold change ratio) display differences between the systemic pot in the treatment treated with FOL or FA as compared to their corresponding control for each cultivar. Hierarchical clustering was performed using the Euclidean distance and the complete clustering method. The dendogram linkages of the genus are not phylogenetic, but based on the log_2 fold change ratio of the genus. Colored bars at the right panel represent the taxonomic affiliations of the genera at the phylum level. S, susceptible cultivar D72; R, resistant cultivar Z19. **b** Venn diagrams display the number of shared and unique genus that increased or decreased by FOL or FA, in each cultivar. FOL, *F. oxysporum* f. sp. *lycopersici*; FA, fusaric acid. * represents statistically significant differences (two-sided Wald test, Benjamini-Hochberg adjusted $P < 0.01$).

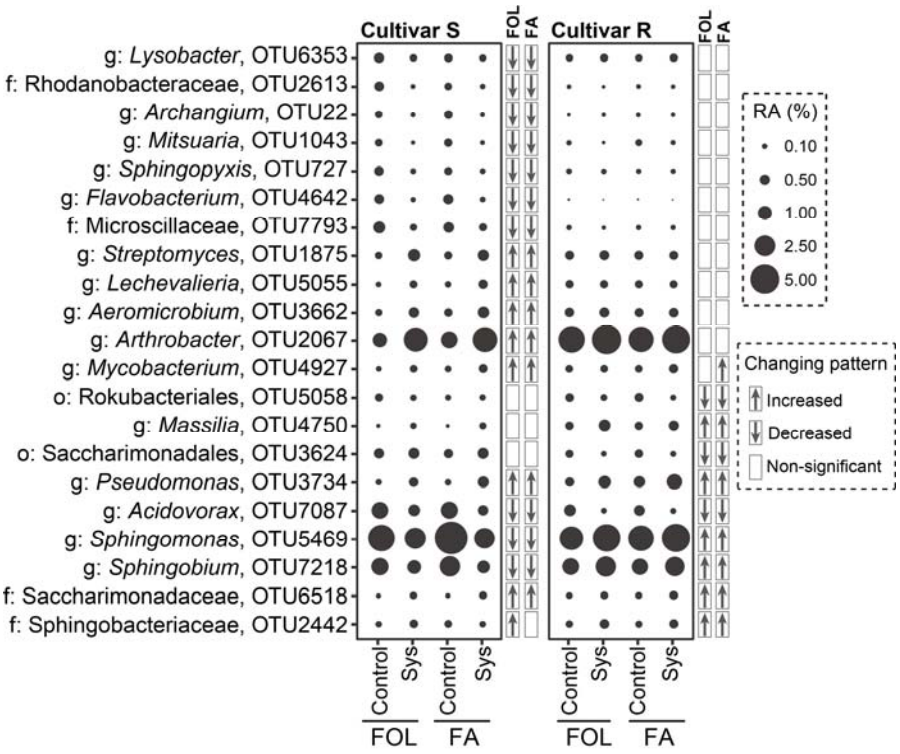

Supplementary Fig. 6 Relative abundance (RA) of OTUs altered by both FOL and FA. The panel displays OTUs that increased or decreased in relative abundance in the experiment used to test the effects of FOL and FA on the tomato rhizosphere microbiota. Taxonomic affiliation of the OTU: f, family; o, order; g, genus. The arrows on the right side of the panels indicated the changing pattern of each OTU. S, susceptible cultivar D72; R, resistant cultivar Z19; FOL, *F. oxysporum* f. sp. *lycopersici*; FA, fusaric acid; Sys, systemic.

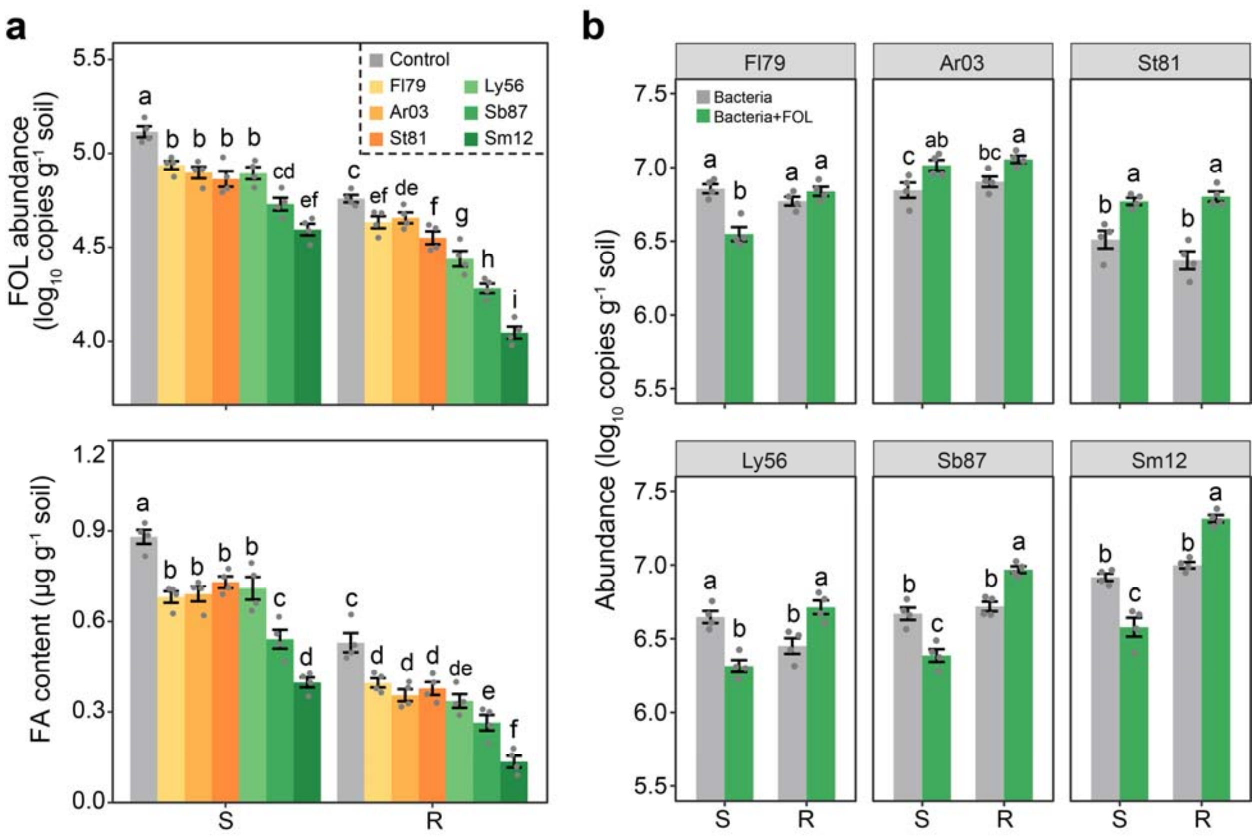

Supplementary Fig. 7 Interaction between FOL and each bacterial isolate in the split-root system. a Effects of bacterial isolates on FOL abundance and FA content in tomato rhizosphere. In the split-root system, one part of the root system was inoculated with FOL, and the other part was inoculated with each bacterial isolate or untreated. FOL abundance and FA content in the rhizosphere of tomato from the pot inoculated with FOL was quantified. S, susceptible cultivar D72; R, resistant cultivar Z19. **b** Effects of FOL infection on the root colonization by each bacterial isolate inoculated as a single taxon. In the split-root system, one part of the root system was inoculated with each bacterial isolate, and the other part was inoculated with FOL or untreated. Bacterial abundance in the rhizosphere of tomato from the pot inoculated with bacteria was quantified. FOL, *F. oxysporum* f. sp. *lycopersici*; FA, fusaric acid. Data are shown as mean ± SEM (n=4). Different letters represent significant differences between treatments (Tukey's HSD test; $P < 0.05$).

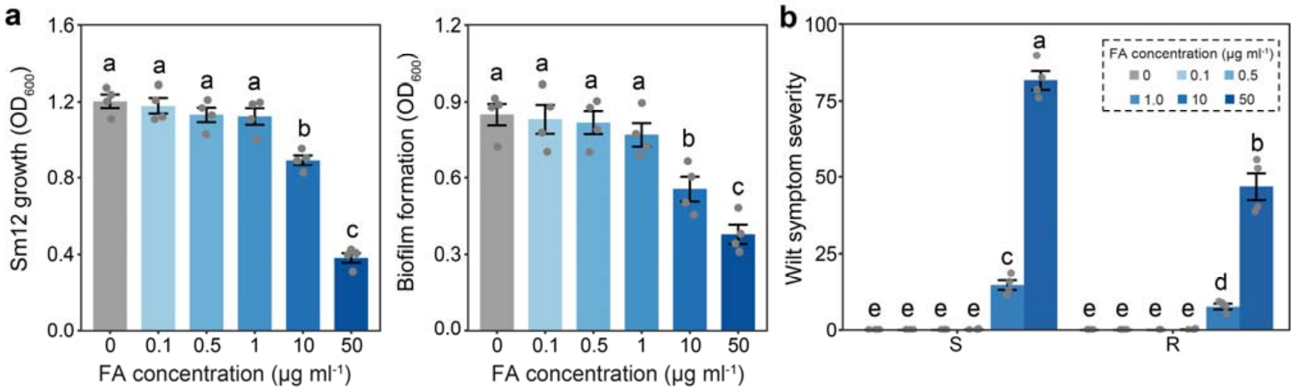

Supplementary Fig. 8 Toxic effects of FA on bacteria Sm12 and tomato plants. a Effects of FA on the growth and biofilm formation of Sm12. **b** Effects of FA on wilt symptom of tomato plants. S, susceptible cultivar D72; R, resistant cultivar Z19; FA, fusaric acid; OD, optical density. Data are shown as mean ± SEM (*n*=4). Different letters represent significant differences between treatments (Tukey's HSD test; $P < 0.05$).

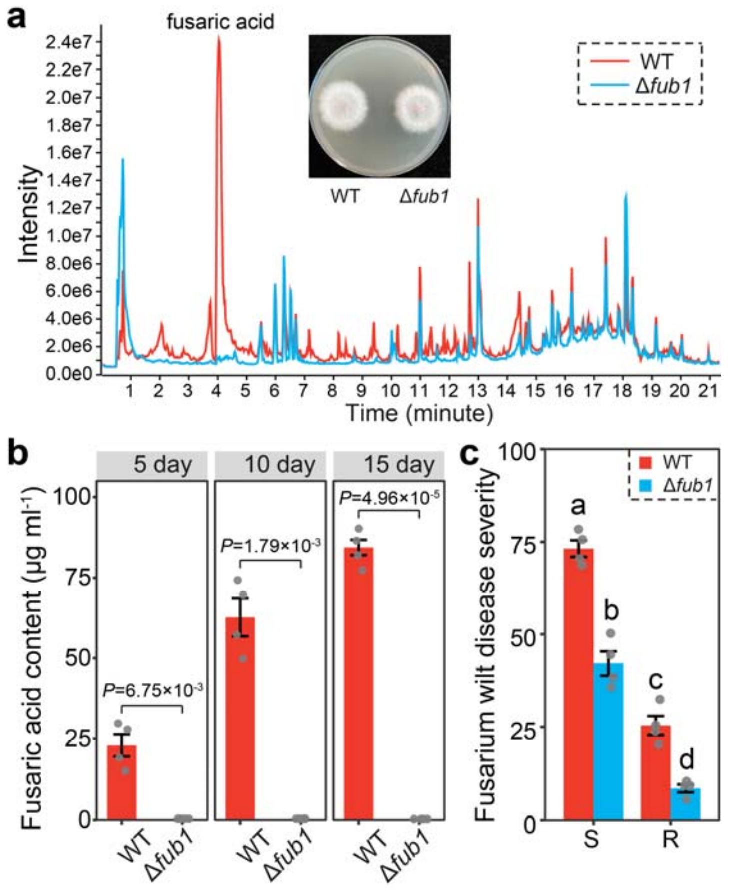

Supplementary Fig. 9 Characteristics of the *Δfub1* mutant. a The HPLC-MS chromatograms for culture extracts of the wild-type FOL (WT) and the Δ*fub1* mutant. The inserted photo shows the colonies of the wild-type FOL and the Δ*fub1* mutant grown on potato dextrose agar. The experiment was repeated six times with similar results. **b** FA production by the wild-type FOL and the Δ*fub1* mutant when grown in potato dextrose medium. **c** Pathogenicity of the wild type FOL and the Δ*fub1* mutant in tomato plants. S, susceptible cultivar D72; R, resistant cultivar Z19. For **b** and **c**, data are shown as mean ± SEM (n=4). For **b**, different letters represent significant differences between treatments (Tukey's HSD test; $P < 0.05$). For **c**, P values were determined through two-sided Welch's t-tests.

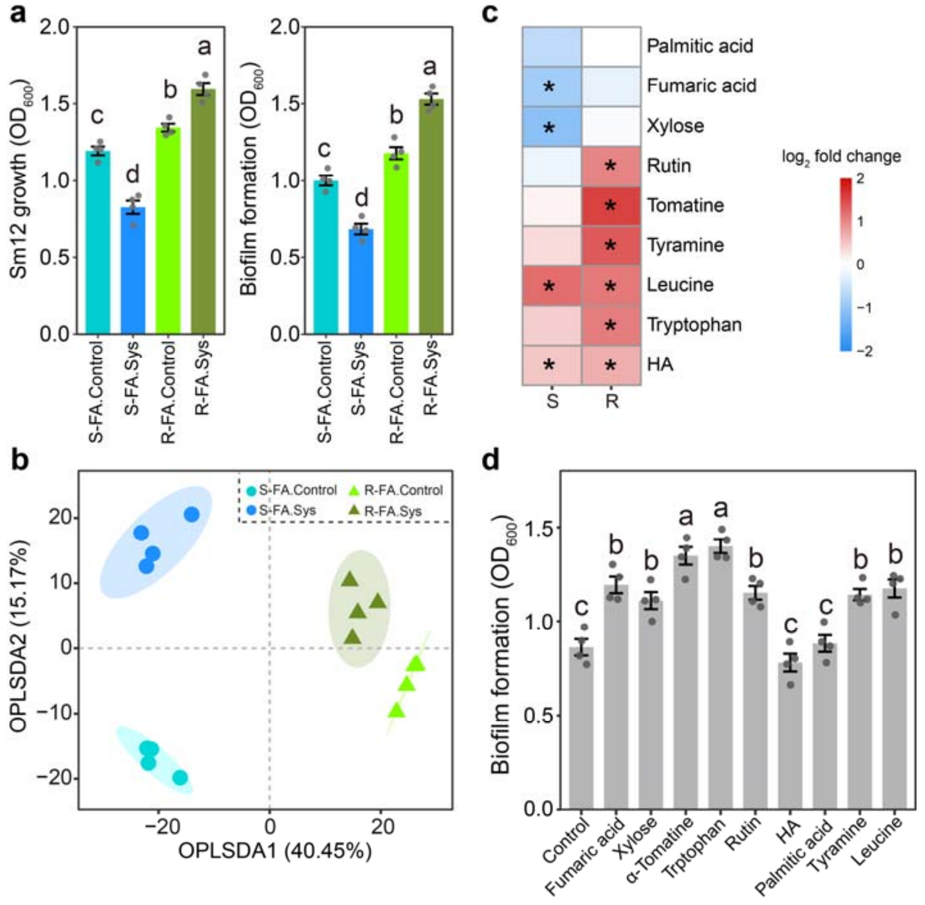

Supplementary Fig. 10 Effects of FA on tomato root exudates and effects of pre-identified compounds on the biofilm formation of Sm12. a Effects of tomato root exudates on the growth and biofilm formation of Sm12. Tomato root exudates were collected from the systemic pot of FA-treated plants (FA.Sys) and from the untreated control (FA.Control). S, susceptible cultivar D72; R, resistant cultivar Z19. **b** Orthogonal partial least squares- discriminant analysis (OPLS-DA) of metabolites in tomato root exudates. **c** Heatmap depicting the changing patterns of metabolites in tomato root exudates affected by FA amendment. *indicates significant altered metabolites with variable importance of projection > 1, $\log_2$ fold change > 1 and Benjamini-Hochberg adjusted $P < 0.01$ (two-sided Wald test). **d** Effects of pre-identified compounds on the growth and biofilm formation of Sm12. HA, 2-Hydroxyglutaric acid; FA, fusaric acid; OD, optical density. For **a** and **d**, data are shown as mean ± SEM (n=4). Different letters represent significant differences between treatments (Tukey's HSD test; $P < 0.05$).

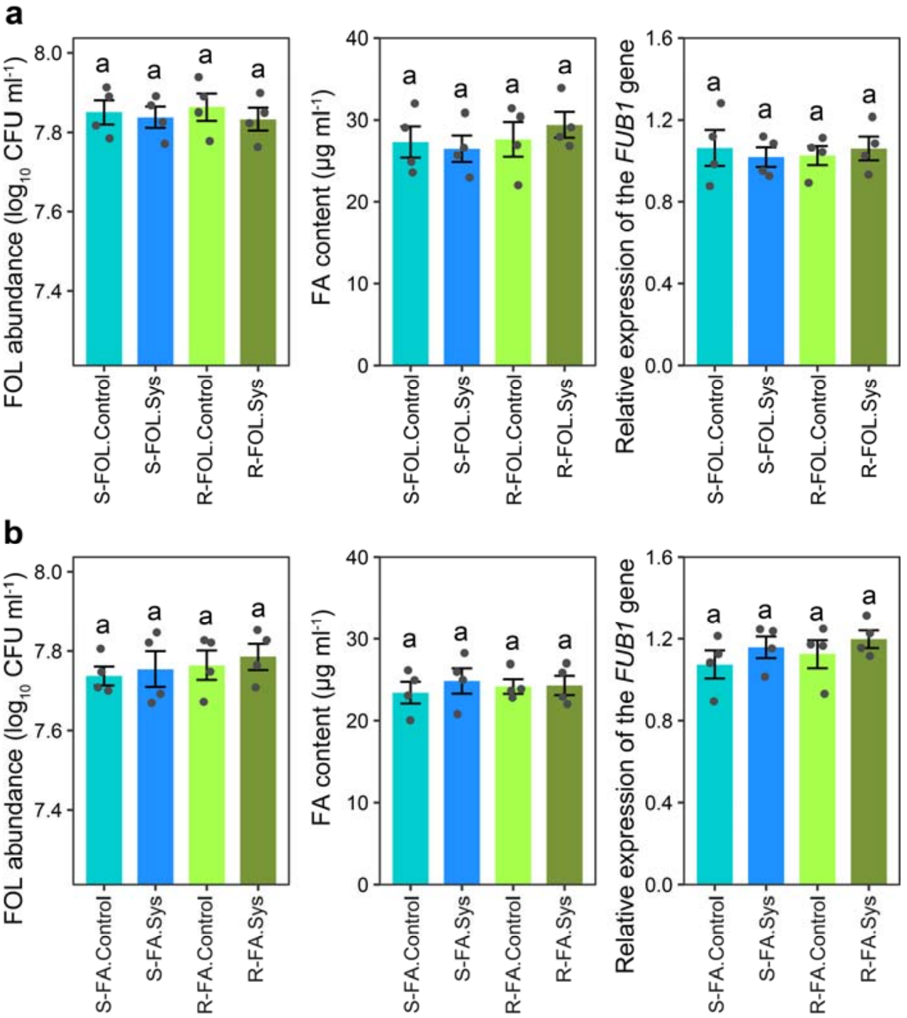

Supplementary Fig. 11 Effects of tomato root exudates on FOL growth and FA production. a Effects of root exudates of tomato plants inoculated with FOL or not on FOL growth, FA production and the expression of the FA biosynthetic gene 1 (*FUB1*). Tomato root exudates were collected from the systemic pot of FOL-inocluated plants (FOL.Sys) and from the untreated control (FOL.Control). S, susceptible cultivar D72; R, resistant cultivar Z19. **b** Effects of root exudates of tomato plants treated with FA or not on FOL growth, FA production and the expression of the *FUB1* gene. Tomato root exudates were collected from the systemic pot of FA-treated plants (FA.Sys) and from the untreated control (FA.Control). CFU, colony forming units; FA, fusaric acid; FOL, *F. oxysporum* f. sp. *lycopersici*; FA, fusaric acid. Data are shown as mean ± SEM (n=4). Different letters represent significant differences between treatments (Tukey's HSD test; $P < 0.05$).

Supplementary Table 1. List of PCR primers and probes used in this study

Target group/use	Primer name and sequence (5´-3´)	Reference
AOS gene of tomato	forward, CCGGCGGGAAGATCACGATG<br>reverse, TCGAAAACGGCGTCGTGTGA	Lee et al. (2021) [1]
PR1a gene of tomato	forward, CTGGTGCTGTGAAGATGTGG<br>reverse, TGACCCTAGCACAACCAAGA	Zhou et al. (2023) [2]
ACTIN gene of tomato	forward, TGAATGCACGGTAGCAAACAACAGATT<br>reverse, AATGCATCAGGCACCTCTCAAGTAT	Zhou et al. (2023) [2]
Bacteria	338F, ACTCCTACGGGAGGCAGC<br>806R, GGACTACHVGGGTWTCTAAT	Caporaso et al. (2012) [3]
Bacteria	27F, AGAGTTTGATC(A/C)TGGCTCAG<br>1492R, TACGG(C/T)TACCTTGTTACGACTT	Heuer et al. (1997) [4]
Flavobacterium sp.	859-RT-F2, ACAATCCATAGGACCGTCATCCTG<br>859-RT-R2, TGAGACACGGACCAGACTCCTAC	Yin et al. (2013) [5]
Arthrobacter sp.	Art627F, GATCTGCGGTGGGTACGG<br>Art985R, CGGTTCATGTCAAGCCTT	Dsouza et al. (2015) [6]
Sphingomonas sp.	Sphingo108F, GCGTAACGCGTGGGAATCTG<br>Sphingo420R, TTACAACCCTAAGGCCTTC	Leys et al. (2004) [7]
Streptomyces sp.	Strep661f, GTAGGGGAGATCGGAATT<br>Sterp1218r, AGCACGTGTGCAGCCCAA	Inbar et al. (2005) [8]
Sphingobium sp.	SGB.5F, ACAGTACCGGGAGAATAAGCTC<br>SBG.5R, CAAGCAATCCAGTCTCAAAGGCTA	Jones et al. (2011) [9]
Lysobacter sp.	LysoF1, CGGGTTGTAAAGCWCTTTTGTCC<br>LysoR1, GAAGTTAGCCGGTGCTTATTCTTCC	Iwasaki et al. (2020) [10]
Fusarium oxysporum f. sp. *lycopersici*	FOL3f, AACCTGGTACCCCGAATCG<br>FOL3r, GAGAGTGCCCGCCATCTG<br>TaqMan Probe, ATCGCCTGGGACCTACGATTATTTCGC	Huang et al. (2016) [11]
Upstream of the *FUB1* gene	*fub*1-F1, GCTACCCATATAACACAACGG<br>*fub*1-R1, CTTGCTTTGGATTTGACTCTG	López-Díaz et al. (2018) [12]
Downstream of the *FUB1* gene	*fub1*-F2, TCTATGTTTTATTTCACTCAGGG<br>*fub1*-R2, GCGTTCCTCTTCACTAAATTAT	
The hygromycin B phosphotransferase cassette	*fub*1-*hph*-F, TTCTTCAGAGTCAAATCCAAAGCAAGCGAGACCTAATACAGCCCCTA<br>*fub*1-*hph*-R, ATGCCCTGAGTGAAATAAAACATAGACCTGTGCATTCTGGGTAAACG	
Fusion PCR	*fub*1-F1n, AATACTCTCCTCTAAGCAGCC<br>*fub*1-R2n, AAATCCCAAACCCCCTCAATC	
qRT-PCR for *FUB1* gene of FOL	*fub1*-F3, GGGTTTGAGTTGTGCTGAGG<br>*fub1*-R3, AGAACGCTCATTATACTTGCTG	
qRT-PCR for *Actin* gene of FOL	*act*-F, ATGTCACCACCTTCAACTCCA<br>*act*-R, CTCTCGTCGTACTCCTGCTT	Ruiz et al. (2016)[13]

Supplementary references

1. Lee, S.-M., Kong H. G., Song G. C. & Ryu C.-M. Disruption of Firmicutes and Actinobacteria abundance in tomato rhizosphere causes the incidence of bacterial wilt disease. *ISME J.* **15**, 330-347 (2021).
2. Zhou, X. et al. Interspecific plant interaction via root exudates structures the disease suppressiveness of rhizosphere microbiomes. *Mol. Plant* **16**, 849-864 (2023).
3. Caporaso, J. G. et al. Ultra-high-throughput microbial community analysis on the Illumina HiSeq and MiSeq platforms. *ISME J.* **6**, 1621-1624 (2012).
4. Heuer, H., Krsek M., Baker P., Smalla K. & Wellington E. M. Analysis of actinomycete communities by specific amplification of genes encoding 16S rRNA and gel-electrophoretic separation in denaturing gradients. *Appl. Environ. Microbiol.* **63**, 3233-3241 (1997).
5. Yin, C. et al. Role of bacterial communities in the natural suppression of *Rhizoctonia solani* bare patch disease of wheat (*Triticum aestivum* L.). *Appl. Environ. Microbiol.* **79**, 7428-7438 (2013).
6. Dsouza, M., Taylor M. W., Turner S. J. & Aislabie J. Genomic and phenotypic insights into the ecology of *Arthrobacter* from Antarctic soils. *BMC Genomics* **16**, 36 (2015).
7. Leys, N. M. E. J. et al. Occurrence and phylogenetic diversity of *Sphingomonas* strains in soils contaminated with polycyclic aromatic hydrocarbons. *Appl. Environ. Microbiol.* **70**, 1944-1955 (2004).
8. Inbar, E., Green S. J., Hadar Y. & Minz D. Competing factors of compost concentration and proximity to root affect the distribution of streptomycetes. *Microb. Ecol.* **50**, 73-81 (2005).
9. Jones, M. D., Singleton D. R., Sun W. & Aitken M. D. Multiple DNA extractions coupled with stable-isotope probing of anthracene-degrading bacteria in contaminated soil. *Appl. Environ. Microbiol.* **77**, 2984-2991 (2011).
10. Iwasaki, Y., Ichino T. & Saito A. Transition of the bacterial community and culturable chitinolytic bacteria in chitin-treated upland soil: from *Streptomyces* to methionine-auxotrophic *Lysobacter* and other genera. *Microbes Environ.* **35**, ME19070 (2020).
11. Huang, C.-H., Tsai R.-T. & Vallad G. E. Development of a TaqMan real-time polymerase chain reaction assay for detection and quantification of *Fusarium oxysporum* f. sp. *lycopersici* in soil. *J. Phytopathol.* **164**, 455-463 (2016).
12. López-Díaz, C. et al. Fusaric acid contributes to virulence of *Fusarium oxysporum* on plant and mammalian hosts. *Mol. Plant Pathol.* **19**, 440-453 (2018).
13. Bravo Ruiz, G., Di Pietro A. & Roncero M. I. G. Combined action of the major secreted exo- and endopolygalacturonases is required for full virulence of *Fusarium oxysporum*. *Mol. Plant Pathol.* **17**, 339-353 (2016).
